# Integrin β1 is a key determinant of the expression of angiotensin-converting enzyme 2 (ACE2) in the kidney epithelial cells

**DOI:** 10.1016/j.ejcb.2023.151316

**Published:** 2023-04-18

**Authors:** Md Saimon Mia, Delowar Hossain, Emerson Woodbury, Sean Kelleher, Riya. J. Palamuttam, Reena Rao, Preston Steen, Yagna PR Jarajapu, Sijo Mathew

**Affiliations:** a Department of Pharmaceutical Sciences, School of Pharmacy, North Dakota State University, Fargo, ND, USA; b Vanderbilt University Medical Center, Nashville, TN, USA; c Kidney Institute, University of Kansas Medical Center, Kansas City, KS, USA; d Sanford Health Roger Maris Cancer Center, Fargo, ND, USA

**Keywords:** Integrin, ACE2, Renal epithelial cells

## Abstract

The expression of the angiotensin-converting enzyme 2 (ACE2) is altered in multiple chronic kidney diseases like hypertension and renal fibrosis, where the signaling from the basal membrane proteins is critical for the development and progression of the various pathologies. Integrins are heterodimeric cell surface receptors that have important roles in the progression of these chronic kidney diseases by altering various cell signaling pathways in response to changes in the basement membrane proteins. It is unclear whether integrin or integrin-mediated signaling affects the ACE2 expression in the kidney. The current study tests the hypothesis that integrin β1 regulates the expression of ACE2 in kidney epithelial cells. The role of integrin β1 in ACE2 expression in renal epithelial cells was investigated by shRNA-mediated knockdown and pharmacological inhibition. *In vivo* studies were carried out using epithelial cell-specific deletion of integrin β1 in the kidneys. Deletion of integrin β1 from the mouse renal epithelial cells reduced the expression of ACE2 in the kidney. Furthermore, the downregulation of integrin β1 using shRNA decreased ACE2 expression in human renal epithelial cells. ACE2 expression levels were also decreased in renal epithelial cells and cancer cells when treated with an integrin α2β1 antagonist, BTT 3033. SARS-CoV-2 viral entry to human renal epithelial cells and cancer cells was also inhibited by BTT 3033. This study demonstrates that integrin β1 positively regulates the expression of ACE2, which is required for the entry of SARS-CoV-2 into kidney cells.

## 1. Introduction

Angiotensin-converting enzyme 2 (ACE2) is a secreted carboxypeptidase that converts Ang II to Ang 1–7 and Ang I to Ang 1–9 (Tipnis et al., 2000). In the kidney, this enzyme is principally expressed in the proximal tubule’s epithelial cells and podocytes (Hamming et al., 2004; Lely et al., 2004; Ye et al., 2004). ACE2 expression in the proximal tubules of the kidneys is altered in multiple diseases including diabetic nephropathy, hypertension, and chronic kidney diseases (Soler et al., 2013). The association between ACE2 expression and the progression of kidney diseases suggests that manipulation of this expression may have therapeutic potential. The importance of communication from the extracellular matrix (ECM) to the proximal tubular epithelial cells on ACE2 levels is not known. We hypothesize that the signaling by adhesion molecules from the ECM is crucially important for ACE2 expression in the tubular epithelial cells.

Integrins are transmembrane cell surface adhesion receptors that mediate the communication of cells with the immediate microenvironment. These heterodimeric receptors have noncovalently associated α and β subunits and are present in almost all cell types. The various integrin αβ complexes bind to specific extracellular matrix proteins. Integrins are principally responsible for cell-ECM interactions, cell signaling, epithelial cell polarization, differentiation, migration, and proliferation(Bennett, 2015; Hynes, 1992; Iwamoto and Calderwood, 2015). The expression of many different integrin subunits are altered during tumorigenesis, cancer progression, and fibrosis(Binkley et al., 2004; Korhonen et al., 1994; Linder et al., 2001; Meyer et al., 1999; Raab-Westphal et al., 2017; Shimoyama et al., 1995; Weinel et al., 1992).

Apart from mediating the cell-basement membrane interactions, integrins are also reported to be important for viral entry. The association of a virus with an integrin molecule induces quaternary structural changes resulting in the clustering of integrin subunits and promotion of viral endocytosis(Stewart and Nemerow, 2007). Reciprocally, the interactions of viruses with integrins also induce viral surface protein conformational changes exposing the other domains(Lopez and Arias, 2004). The specificity of integrin complexes in determining virus entry has also been demonstrated. Various adenoviruses utilize integrin α5β1and αvβ1 for cell entry(Davison et al., 1997; Li et al., 2001). The rotaviruses spike protein, VP5, bind with integrins α2β1 to facilitate internalization(Graham et al., 2006). The members of the Picornaviridae family, including echovirus-1 (EV1), utilize the α2β1 integrin to promote cell entry (King et al., 1997). In addition, integrin β1 also mediates the internalization of mammalian reoviruses(Maginnis et al., 2006). These studies underscore the critical role of integrin β1 in mediating viral entry to cells.

Severe Acute Respiratory Syndrome Corona Virus 2 (SARS-CoV-2) caused a global pandemic and led to more than a million deaths in the USA. The primary pathologic mechanism in human infections is mediated by the binding of the structural spike protein of the virus to ACE2 (Shang et al., 2020a; Zhou et al., 2020). The current proposed model for this infection implicates ACE2 binding to the RBD domain of spike proteins, and then undergoing endocytosis by the cells. A new binding site in the spike protein was identified recently which has the potential to bind to integrin, suggesting the important role of integrin receptors in facilitating viral entry(Sigrist et al., 2020). The viral spike proteins bind to integrin receptors through a conserved RGD motif lies the receptor binding domain that facilitates the viral endocytosis. A recent study suggested the use of integrin α5β1 inhibitor ATN-161 for the peptide-based masking and neutralization of the SARS-CoV-2 spike protein endocytosis highlighting the importance of integrin β1 in assisting viral entry to the host cells(Rabbani et al., 2021). A recent 3D homology structural model revealed the SARS-CoV-2 spike protein’s RGD domain lies near to the ACE2 binding domain. Binding of ACE2 induces conformational changes and expose the RGD domain in the spike proteins and facilitates viral endocytosis(Yan et al., 2020a). Previous studies have shown that integrin β1 has a role in regulating the localization of ACE2(Clarke et al., 2012; Lin et al., 2004). It is not known what, if any, role integrin β1 plays in ACE2 expression and functions in cells.

In this study, we examined the quantitative relation between integrin β1 and ACE2 in renal epithelial cells, and whether decreased integrin β1 in cells reduces ACE2-mediated endocytosis of SARS-CoV-2 spike protein. Integrin β1 deletion/downregulation decreased the expression of ACE2 in mouse renal epithelial cells, normal renal epithelial cells, and renal cancer cells. Furthermore, a pharmacological integrin α2β1 inhibitor decreased ACE2 expression and the internalization of SARS-CoV-2 spike protein in kidney epithelial cells and cancer cells indicating the functional importance of the ACE2-integrin β1 complex. These findings reveal a new role for integrin β1 in regulating ACE2 levels in the kidneys and is important in multiple chronic diseases.

## 2. Materials and methods

### 2.1. Animal studies

All animal breeding, housing, and experimental approaches were approved by the Institutional Animal Care and Use Committee (IACUC) at NDSU. C57BL/6 wild type mice were obtained from Jackson Laboratory (Harbor, ME, USA). To generate kidney epithelial specific integrin β1 KO mice, ITGB^fl/fl^ mice were crossed with γGT-Cre mice (Jackson Laboratory, Harbor, ME, USA). The gene deletion was confirmed by PCR based analysis of genomic DNA isolated from the tail. PCR amplification the following primers were used: Integrin β1, forward 5^′^- CGGCTCAAAGCAGAGTGTCAGTC –3^′^, reverse 5^′^ - CCACAACTTTCCCAGTTAGCTCTC-3^′^; γgt-Cre, 5^′^-GCTCTTGGGAGAAGTCATGC-3^′^, and 5^′^- CATGTTTAGCTGGCCCAAAT- 3^′^.

### 2.2. Reagents

HK-2 and Caki-1 cells used in this study were obtained from ATCC. Cell culture media and supplements, Dulbecco’s modified Eagle’s medium (DMEM) (Cat: MT10090CV; Corning), Macoy’s 5 A media, fetal Bovine serum (FBS) (F0926; Millipore Sigma), and antibiotic antimycotic solution (Cat: 30–004-Cl; Corning) were used. Primary antibodies used were integrin β1 (D2E5) Rabbit mAb (Cat: 9699; Cell Signaling); antibody for active integrin β1 12G10 (Cat: MAB2247; Millipore Sigma), integrin β1 (Cat: MABT821; Millipore Sigma), Integrin β1, (Santa Cruz Biotechnology Cat: SC-374430), ACE2 monoclonal antibody (Cat: MA5–32307; Thermo Fisher), GAPDH (Cat: 5174; Cell Signaling). Anti His antibody (Cat: SC53073, Santa Cruz Biotechnology). Spike protein (Cat: 230–20407–200, Ray Biotech) and the Control protein (Cat: 230–20001, Ray Biotech).

### 2.3. Cell culture

HK-2 cells were grown in DMEM media supplemented with 0.05 mg/ml Bovine pituitary extract (BPE) and 5 ng/ml EGF (Cat: 17005–042, GIBCO). Caki-1, cells were cultured in Mcoy’s 5 A media (Cat: MT10090CV; Corning) supplemented with 10% Fetal Bovine Serum (FBS) (Cat: F0926; Millipore Sigma) and 0.1% antibiotic antimycotic solution (Cat: 30–004-Cl; Corning). Both the Cell lines were maintained in a humidified incubator containing 5% CO_2_ at 37 °C. Protein expression was downregulated by shRNA plasmid using lipofectamine 3000 transfection reagent (Cat: L3000015; Fisher Scientific). Diluted lipofectamine 3000 Reagent and plasmid DNA solution were mixed in a 1:1 ratio and incubated for 12 h in serum-free media. After that, cells were moved into the serum-containing medium for 48 h, and clones were selected with puromycin (1 μg/ml) (Cat: L3000015; Fisher Scientific).

### 2.4. Western blotting and qRT-PCR

The kidney cortices of integrin wild-type and knock-out mice were collected. These tissues were chopped into small pieces and homogenized for tissue lysate and for total RNA extraction. For tissue lysate, kidney cortices were homogenized in RIPA Buffer and protease and Phosphatase mix. (RIPA buffer composition: 150 nM NaCl, 50 nM Tris, 1% Triton-X-100, 0.5% Sodium deoxycholate, 0.1% SDS). Homogenate was centrifuged at 14000 rpm for 10 min. BCA protein assay kit was used to determine protein concentration in the supernatant. Western blotting for integrin β1 Integrin β1, (Santa Cruz Biotechnology Cat: SC-374430) 1:1000 dilution and incubated overnight at 4 °C. After treating with HRP conjugated secondary antibody these blots were developed by chemiluminescence. Total RNA was extracted by using trizol-chloroform method (Mia et al., 2021). RNA pellets were washed with 75% ethanol and dissolved in 70 μl RNAse-free water. RNA concentration and purity were determined with Nano-drop. Samples were stored at −80 °C until further experiments.

For cell lysates, 70–80% confluence cells were used to prepare cell lysate. Lysis buffer cocktail containing cell lysis buffer (20X) (Cat: 9803S; Bio-rad) and protease & phosphatase inhibitor cocktail (100X) (Cat: 1861281; Thermo Fisher Scientific) were used. Cells scraped by cell scraper, and lysis buffer was mixed for 30 min at 4 °C. Centrifuged at 15000 rpm for 15 min at 4 °C, and the supernatant was collected. Bicinchoninic acid (BCA) Protein Assay Kit (23225; Thermo Fisher Scientific) was used to determine the protein concentration. For Kidney samples, homogenized samples were centrifuged at 14000 rpm for 10 min at 4 °C and the supernatant was collected. For gel electrophoresis, samples were mixed with 2x SDS sample buffer, and the proteins were resolved using Bio-Rad 4–20% precast polyacrylamide gel, 8.6 × 6.7 cm (4561093; Bio-Rad). Protein samples were transferred to 0.2 μm polyvinylidene difluoride (PVDF) membranes (88520; Thermo Fisher Scientific). Membranes were blocked with 5% fat-free milk (Cat: sc-2325; Santa Cruz Biotechnology) in Tris buffer saline (TBS) with 0.1% Tween-20 (Cat: sc-29113; SCB) (TBST) for 1 h. After that, these membranes were washed three times with TBST and incubated with the primary antibodies at 4 °C. Integrin β1 (Cell Signaling Cat: 9699, 1:1000), ACE2 antibody (Thermo Fisher Cat: MA5–32307, 1:1000), anti-His antibody (Santa Cruz Biotechnology (SC53073, 1;1000) 5% Bovine Serum Albumin (BSA) (Cat: BP1600; Fisher Scientific) in Tris buffer saline (TBS) with 0.1% Tween-20 (TBST) (Cat: L3000015; Fisher Scientific) was used to dilute primary and secondary antibody. Enhanced chemiluminescence (ECL) or ECL plus was used to visualize in the Western blotting detection system (RPN2106; GE Healthcare). GAPDH was used as the loading control. Quantification was done using imageJ software (V:1.53 C NIH).

Total RNA was isolated from kidneys and cells using RiboZol RNA Extraction Reagent (N580; VWR) following the manufacturer’s protocol. cDNA synthesis was carried out using OneScript cDNA synthesis kit (Cat: G-234; abmgoods). Quantitative Real-time PCR (qRT-PCR) was performed with Brightgreen 2X qPCR MasterMix-No dye (MasterS; abmgoods). 18 S was used as gene control. Fold changes in mRNA levels were calculated using 2^−ΔΔCt^. For each data point, experiments were conducted in triplicate. Primer sequences ITGB1 forward: 5^′^-CGG CTC AAA GCA GAG TGT CAG TC-3^′^, reverse: 5^′^-CCA CAA CTT TCC CAG TTA GCT CTC-3^′^. ACE2 forward: 5^′^- CAT TGG AGC AAG TGT TGG ATC TT-3^′^; Reverse 5^′^-GAG CTA ATG CAT GCC ATT CTC A-3^′^. 18 S Forward5^′^- GTA ACC CGT TGA ACC CCA TT –3 and reverse 5^′^- CCA TCC AAT CGG TAG TAG CG –3^′^.

### 2.5. Flow cytometry

The surface expression of integrin β1 was investigated by Flow cytometry (BD FACS, Melody). 70–80% of confluent cells were detached using trypsin. 200,000 cells were stained with integrin β1 antibody (Cat: Abcam, ab179471) 1:100 dilution at room temperature for 1 hr in DMEM F-12 media. After 3 washes, cells were counterstained with BV421-conjugated anti-rabbit secondary antibody (Dilution: 1: 1000; Cat: 565014, BD Bioscience) for 30 min at room temperature. The cells were washed 3 times and suspended in 500uL using DMEMF-12 media without phenol red. 30,000 cells were recorded for the fluorescence intensity by flow cytometer. Cells treated with BV421-conjugated anti-rabbit secondary antibody (Dilution: 1: 1000; Cat: 565014, BD Bioscience) secondary antibody were used as a negative control. Data were analyzed using FLOWJOv10. Median fluorescence intensity was determined and used to compare the protein expression between cells.

### 2.6. TCGA data analysis

TCCGA data was accessed and downloaded through the Xena Genome Browser (University of California Santa Cruz). (Goldman et al., 2020).

### 2.7. Immunofluorescence

Paraffin-embedded sections of the kidney were used for immunohistochemistry of integrin β1 and ACE-2. Sections were deparaffinized using xylene and dehydrated using ethanol (90%,80%,70%, and 60%) and rehydrated with deionized water. Antigen retrieval was carried out by using Tris-EDTA buffer pH 9.0 for 20 min. Sections were blocked with blocking buffer Santa Cruz (Cat: sc-516214) overnight at 4 °C. Incubated with primary antibody 1:200 overnight at 4 °C (Integrin β1, Cat: ab179471) (ACE2, Cat: MA532307, Invitrogen). After washing off the primary antibody, incubated with anti-rabbit Alexa Flour 555 (Dilution; 1:1000; Cat: 4413, Cell signaling) secondary antibody for 2hrs at room temperature. Cells were stained with Lotus tetragonolobus Lectin (LTL) fluorescein (Vector laboratories FL-1321) to stain the proximal tubule epithelium for 30 min at room temperature. After washing with blocking buffer, mounted with prolong gold antifade mounting media with DAPI (Cat: p36931, Invitrogen) media and stored at 4 °C until observed under the microscope. Images were obtained using Zeiss LSM 900 confocal microscope.

60–70% of confluence cells were grown on glass coverslips in cell culture media. Cells were rinsed with PBS containing 1.0 mM CaCl_2_ and 0.5 mM MgCl_2_ and fixed with 4% paraformaldehyde (Cat: NC1040701; Fisher Scientific) for 15 min at room temperature. Fixed cells were blocked with BSA for 1 h at room temperature prior to primary anybody treatment. Primary antibody for active integrin β1 (Dilution: 1:250; Cat: MAB2247, clone 12G10; Millipore Sigma) and total integrin β1 (Dilution: 1:250; Abcam, ab179471) was incubated at 4°C. Alexa Flour 488 (Dilution: 1: 1000) and Alexa Flour 555 (Dilution: 1:1000) tagged secondary was used to detect active and total integrin β1, respectively. ProLong^™^ Gold Antifade with DAPI (Cat: P36931; Thermo Fisher Scientific) was used to mount the cells. LSM 900 confocal microscope (Zeiss, Germany) was used to record the fluorescence images. ImageJ software (V:1.53 C NIH) was used to quantify mean area intensity.

### 2.8. BTT 3033 treatment and spike protein internalization

60–70% confluent cells were treated with 140 nM BTT 3033 dissolved in DMSO for 2 h. An equal amount of DMSO was used as a control treatment. After that, cells were washed with PBS, fixed with 4% formaldehyde, and stained with primary and secondary antibodies for immunofluorescent imaging using 40x objective. Confocal images were processed with Zen 3.5 blue with threshold adjusted to black 0, gamma 1 and white 255 in case of HK 2 cells. Similar treatment with BTT 3033 was carried out for Caki-1 cells too. Confocal images were processed with Zen 3.5 blue with threshold adjusted to Black 0, gamma 1, white150. Integrated fluorescence of the cells was normalized to cell spread area and illustrated. Cells were grown to 60–70% confluency in a 12-well plate. Spike protein (Cat: 230–20407–200, RayBiotech) and the Control protein (Cat: 230–20001, RayBiotech) 140 nM of BTT 3033 and an equal volume of DMSO were treated for 2 h. After that, media was removed, and 10uL Spike protein and control proteins were added to DMEM media. After ten minutes, media was removed, and lysis buffer and triazole were used for immunoblotting and total mRNA preparations, respectively. Immunoblotting of the cell lysates after BTT treatment was carried out using Anti His antibody 1:2000 (Cat: SC53073, Santa Cruz Biotechnology) and GAPDH 1:1000 (Cat: 5174; Cell Signaling).

### 2.9. ELISA

To determine the internalization of SARS-CoV-2 spike protein, the Human SARS-CoV-2 RBD ELISA kit (EH492RB; Invitrogen) was used. 50–60% confluent cells in a 12-well plate were treated with 140 nM BTT 3033 and equal volume of DMSO was treated as a control for 2 h. After the treatments, cells were incubated with 10μl of spike protein for 10 min, and cell lysate were prepared using lysis buffer and protease and phosphatase inhibitor cocktail. ELISA for the spike protein was performed using the supplier’s protocol. 100μl cell lysate was added to the pre-coated well and incubated overnight at 4°C with gentle shaking. These wells were washed 4 times with washing buffer, and 100μl biotin conjugate was added and incubated for 1 h. Later, these wells were washed, and 100 μl Streptavidin-HRP conjugated was added, incubated for 45 min at room temperature with gentle shaking. The solution was discarded and wells were washed and incubated for 30 min at room temperature in the dark with TMB substrate. 50μl stop solution was added, and the absorbance was taken at 450 nm. Each sample was triplicated, and a Standard curve was performed with each assay.

### 2.10. Statistical analysis

*In vitro* and in vivo measurement data were presented as the means ± SEM. Paired or unpaired t-test was performed to determine statistical significance. At least three independent experiments were performed for each data. Differences with the minimum of *p* < 0.05 were indicated as statistically significant.

## 3. Results

### 3.1. Downregulation of integrin β1 expression decreased ACE2 expression in HK-2 human renal epithelial cells

**Fig. 1.**
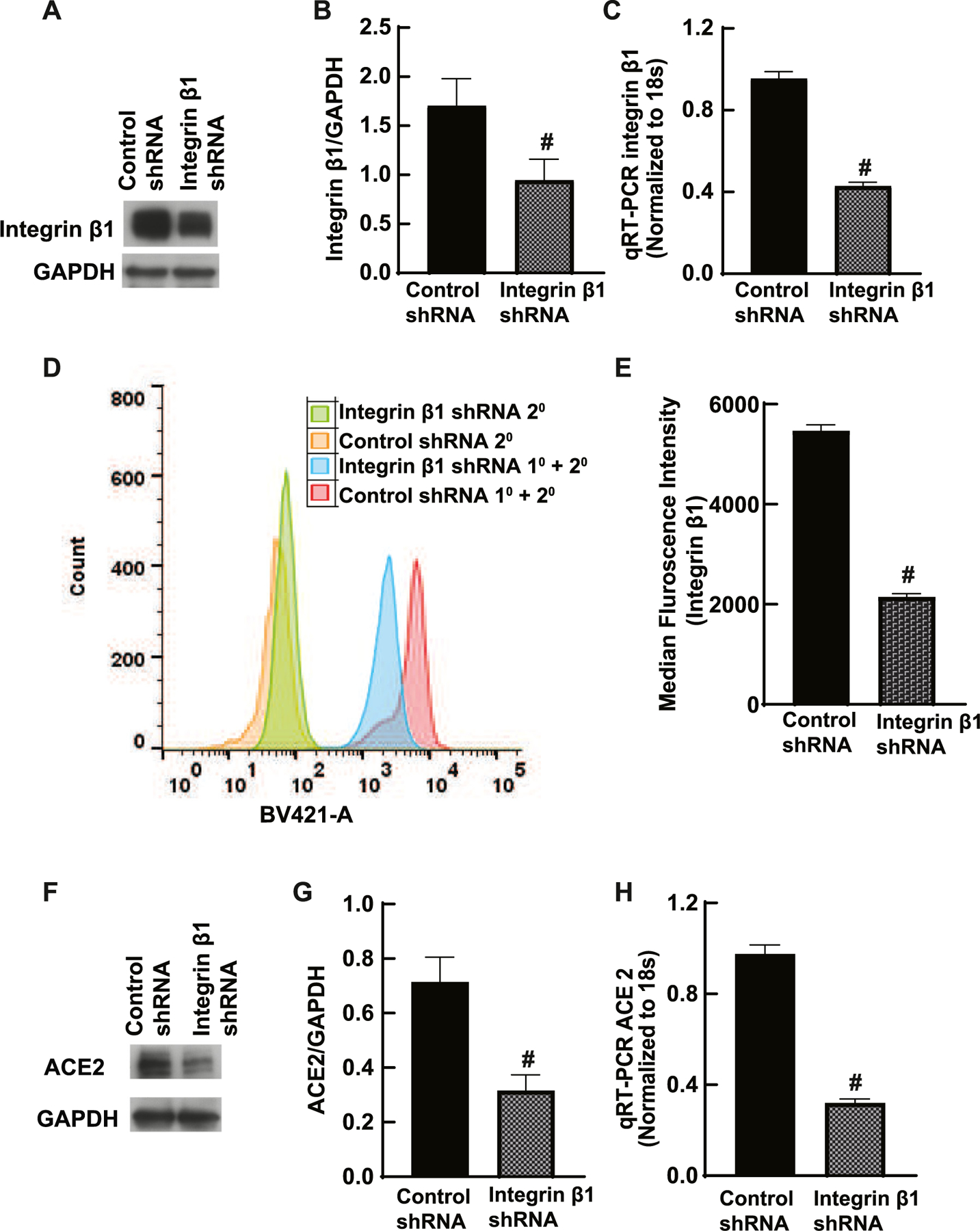
Integrin β1 downregulation decreased ACE2 expression in human renal epithelial cells. A, B) Human renal proximal tubule cell HK-2 was treated with integrin β1 shRNA. The expression of integrin β1 was detected with immunoblotting using integrin β1 antibody (Cell Signaling, 9699) that showed a significant reduction in the presence of integrin β1 shRNA compared to control shRNA treated cells (# *p* < 0.05, n = 3). C) Total mRNA quantified by qRT-PCR showed a significant reduction in integrin β1 shRNA treated cells compared to control shRNA treated cells (# *p* < 0.05, n = 3). D, E) The surface expression of integrin β1 was estimated using Flow cytometry analysis revealed a significant decrease in the median fluorescent intensity. Integrin β1 was stained with primary antibody (Abcam, ab 179471) and BV421-conjugated anti-rabbit secondary antibody (BD Bioscience, 56501) (# *p* < 0.001, n = 3). F, G) ACE2 expression decreased significantly in integrin β1 shRNA-treated HK-2 cells compared to control shRNA-treated cells in western blotting (ACE2 antibody, Thermo Fisher, MA5–32307) (# *p* < 0.05, n = 3). H) ACE2 mRNA as quantified by qRT-PCR also showed a significant reduction in integrin β1 shRNA treated cells compared to control shRNA treated cells (# *p* < 0.05, n = 3).

To determine the role of integrin β1 in ACE2 expression in renal epithelial cells, HK-2 cells were treated with integrin β1 shRNA that reduced protein expression by about 50% compared to control shRNA treated cells (Fig. 1A–E). The decreased expression was confirmed by immunoblotting and qRT-PCR (Fig. 1 A–C). The surface expression of proteins was quantitated using Flow cytometry using 30,000 cells. There was a significant decrease in the median fluorescence intensity (n = 3, *p* < 0.05) in agreement with immunoblotting and qRT-PCR. Approximately 57% decrease in the median fluorescence intensity was observed in integrin β1 shRNA treated cells compared to control shRNA treated cells. Lower integrin β1 protein levels significantly decreased the ACE2 expression in these cells (Fig. 1F–H *p* < 0.05).

### 3.2. BTT 3033 decreased the ACE-2 expression in HK-2 cells

**Fig. 2.**
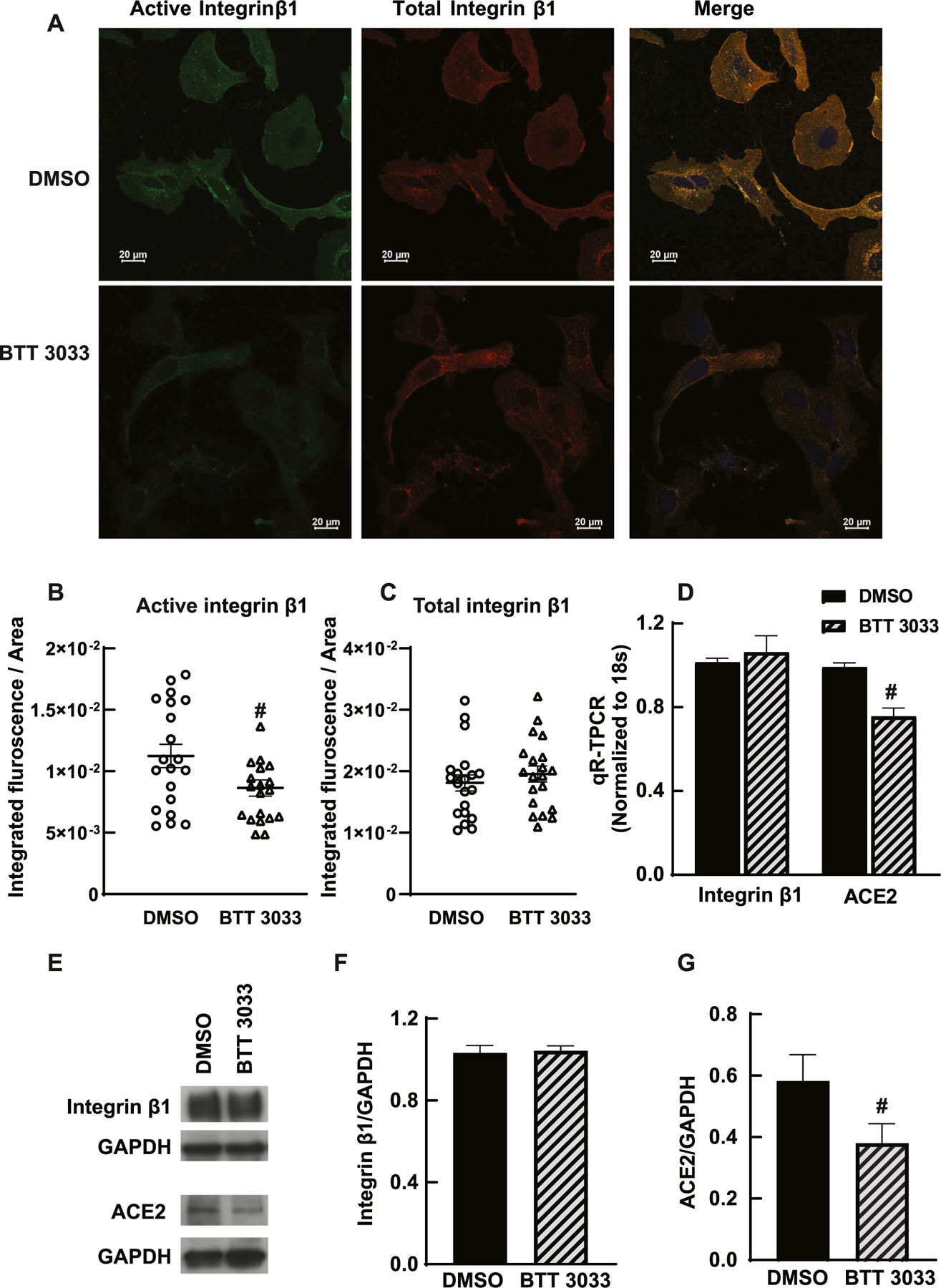
Pharmacological inhibition of integrin β1 using BTT 3033 decreased ACE2 expression in HK-2 cells. A) HK-2 cells were treated with DMSO and integrin α2β1 inhibitor BTT 3033. Treated cells were immune-stained for active integrin β1 (Millipore Sigma, MAB2247, clone 12G10). Total integrin β1 expression was monitored by integrin β1 antibody (Abcam, ab 179471). Scale bar 20 μm. Nucleus was stained with DAPI. B-C) Quantification of active integrin β1 shows a significant decrease in BTT 3033 treatment compared to DMSO in HK-2 Cells (n = 20, * *p* < 0.05) and no change in total integrin β1 expression with BTT 3033 treatment. D) mRNA level of ACE2 in BTT 3033 treated HK-2 cells was significantly lower compared to DMSO treated cells (# *p* < 0.05, n = 3). No significant change in the integrin β1 mRNA level was observed. E, F) Immunoblotting for integrin β1 (9699; Cell Signaling) did not show changes in protein level. G) Immunoblotting confirmed the decreased protein level expression of ACE2 (MA5–32307; Thermo Fisher).

To further confirm the role of integrin β1 in ACE2 expression, the effect of a pharmacological inhibitor of integrin β1, BTT 3033(Nissinen et al., 2012) was studied. HK-2 cell line treated with 140 nM of BTT 3033. The active integrin β1 levels were detected with an antibody, 12G10, that binds specifically to active conformation status. The active integrin β1 levels were decreased by BTT 3033 treatment in HK-2 cells (Fig. 2A–C). Treatment of cells with BTT 3033 did not change the total expression of integrin β1 in the cells (Fig. 2C). The expression of ACE2 decreased in BTT 3033 treated HK-2 cells compared to DMSO treated cells (Fig. 2D). This data suggests that the integrin β1 activation determines the ACE2 expression in renal epithelial cells. Protein and mRNA levels of ACE2 were investigated using qRT-PCR and immunoblotting. Inhibition of integrin α2β1 complex by BTT 3033 significantly decreased ACE2 expression (Fig. 2E–G).

### 3.3. Integrin β1 and ACE2 levels are higher in kidney cancer cells compared to normal kidney cells

**Fig. 3.**
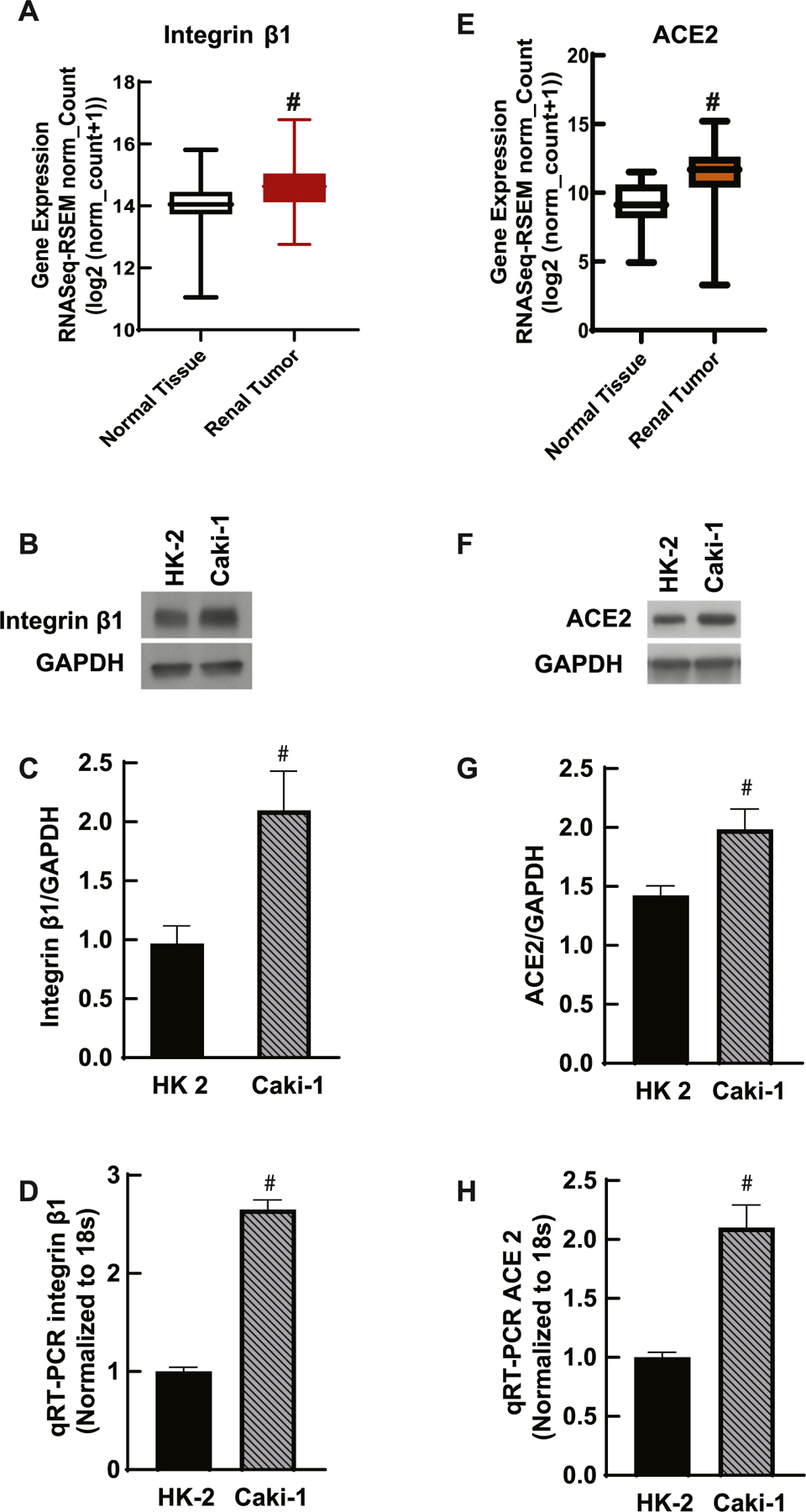
Integrin β1 and ACE2 expression are higher in renal tumors compared to normal control samples. A) Gene expression of integrin β1 in renal tumors was compared to normal tissues using TCGA data and USCF Xena software. Integrin β1 levels were significantly higher in renal tumors (normal n = 160, tumor n = 170, *p* < 0.05). B, C) Immunoblotting of integrin β1 (Cell Signaling, 9699;) in HK-2 and Caki-1 cell line (n = 3) also showed higher integrin β1 expression in renal cancer (Caki-1) cells compared to epithelial cells (HK-2). D) Fold change in integrin β1 mRNA levels by qRT-PCR also shows higher integrin β1 levels in Caki-1 cells compared to renal epithelial cells (HK-2) (n = 3, # *p* < 0.05). E) TCGA data analysis showed significantly higher gene expression of ACE2 in renal tumors compared to normal tissues (normal n = 27, tumor n = 358, *p* < 0.05). F-G) Immunoblotting of ACE2 (Thermo Fisher, MA5–32307) normalized with GAPDH (Cell Signaling, 5174) also showed significantly higher expression of ACE2 in Caki-1 cells compared to renal epithelial cells (HK-2) (# *p* < 0.05, n = 3). H) ACE2 mRNA levels by qRT-PCR also showed higher ACE2 levels in Caki-1 cells compared to renal epithelial cells (HK-2) (# *p* < 0.05, n = 3).

We next examined if integrin β1 regulates ACE2 expression in clear cell renal cell carcinoma (ccRCC) tumors. Although solid tumors are known to have higher integrin β1 expression compared to normal tissues its relation to the expression of ACE2 in these tissues remains unclear. The gene expression of integrin β1 and ACE2 in renal tumors were analyzed using TCGA database. The expression of both proteins showed higher levels in tumor compared to normal tissues (Fig. 3A, E). The level of integrin β1 and ACE2 was compared between renal epithelial cells, HK-2 and ccRCC cells of human origin- the Caki-1 cells. The expression of integrin β1 was significantly higher in Caki-1 (Fig. 3B, C *p* < 0.05). The mRNA also showed higher levels in Caki-1 cells compared to HK-2 cells (Fig. 3D *p* < 0.05). ACE2 expression in Caki-1 was also higher compared to normal epithelial cells (Fig. 3E–H *p* < 0.05).

### 3.4. Integrin α2β1 inhibition decreased ACE2 expression in kidney cancer cells

**Fig. 4.**
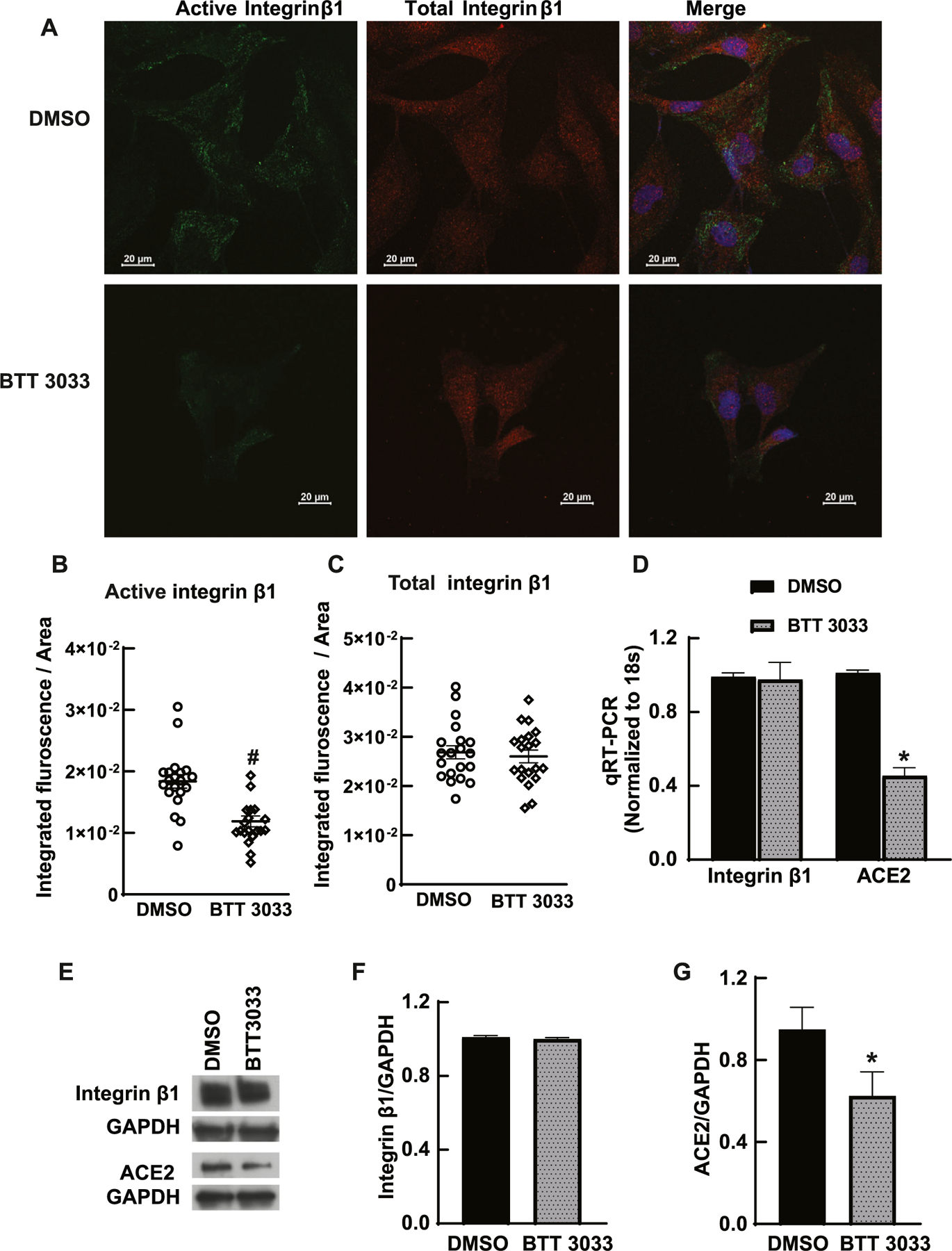
A decrease in the integrin β1 activation lowered ACE2 expression in Caki-1 cells. A) DMSO and BTT 3033 treated Caki-1 cells were stained for active integrin β1 (Millipore Sigma, MAB2247, clone 12G10). Total integrin β1 expression was monitored by integrin β1 antibody (Abcam, ab 179471). Scale bar 20 μm. B-C) Active integrin β1 shows a significant decrease in BTT 3033 treated Caki-1 cells compared to DMSO treated cells (n = 20, # *p* < 0.05) and no change in total integrin β1 expression with BTT 3033 treatment. D) Fold changes in mRNA level of integrin β1 and ACE2 quantified by qRT-PCR showed a significant reduction in ACE2 mRNA levels in BTT 3033 treated cells compared to DMSO treated cells. Integrin β1 mRNA levels did not alter significantly by BTT 3033 treatment. E-G) immunoblotting using integrin β1(Cell Signaling, 9699) and ACE2 (Thermo Fisher, MA5–32307) also confirmed the lower ACE2 levels in BTT 3033 treated cells compared to DMSO treated cells (# *p* < 0.05, n = 3).

When Caki-1 cells were treated with integrin α2β1 inhibitor BTT 3033, integrin β1 activation was lower in BTT 3033 treated cells compared to DMSO treated cells (Fig. 4A). The expressions of integrin β1, and ACE2 were detected by qRT-PCR and immunoblotting. BTT 3033 did not change the expression of integrin β1 (Fig. 4C–F), while the mRNA levels of the ACE2 were significantly decreased in the presence of BTT 3033 compared to DMSO treated cells (Fig. 4D–G). This effect was similar in both HK-2 and Caki-1 cells. This data suggests that integrin α2β1 activation is the major determinant of ACE2 expression in renal cancer cells.

### 3.5. Pharmacological inhibition of integrin α2β1 decreased SARS-CoV-2 spike protein internalization in renal cells

**Fig. 5.**
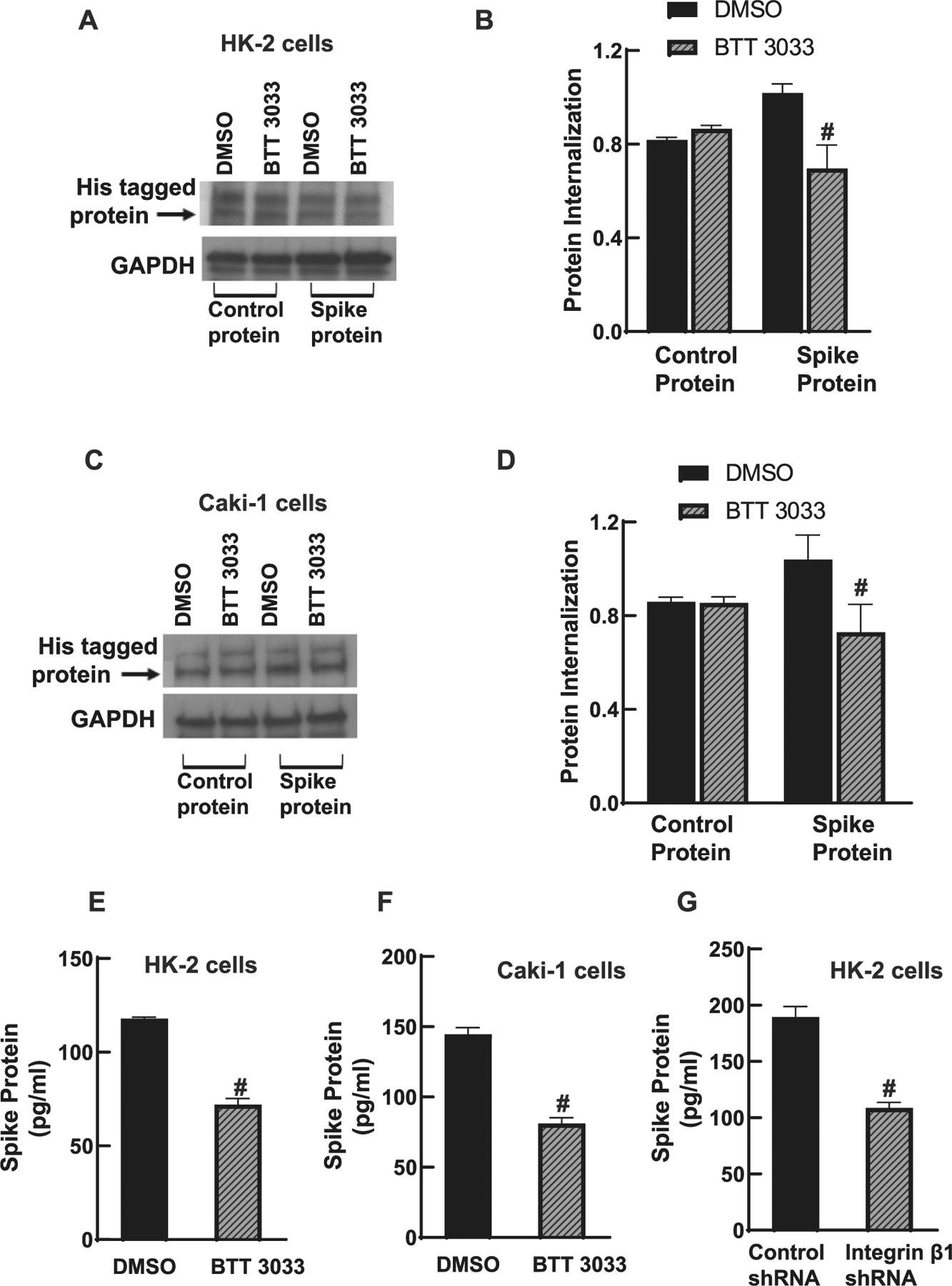
SARS-CoV-2 spike protein internalization decreased in the presence of BTT 3033 in HK-2 and Caki-1 cells. A) Immunoblotting using an anti-His antibody (Santa Cruz Biotechnology, SC53073) for control and SARS-CoV-2 spike protein after 2 h of treatment with DMSO and BTT 3033 in HK-2 cells. GAPDH was used as a loading control (5174; Cell Signaling). B) No change in control protein internalization in DMSO and BTT 3033 treatment groups. Protein quantification shows a significantly lower SARS-CoV-2 spike protein internalization in BTT 3033 treated HK-2 cells (#*p* < 0.05, n = 3) compared to DMSO-treated cells. C) SARS-CoV-2 spike protein internalization was decreased in Caki-1 in the presence of BTT 3033. Caki-1 cell lysates were immunoblotted using an anti-His antibody for control and SARS-CoV-2 spike protein after 2 h of treatment with DMSO and BTT 3033 in HK-2 and Caki-1 cells. D) Normalized protein quantification shows a significantly lower SARS-CoV-2 spike protein internalization in BTT 3033 treated Caki-1 cells (# *p* < 0.05, n = 3) and no change in control protein internalization. E, F) HK-2 and Caki-1cells lysates were analyzed for the internalized spike protein using ELISA. There was a significant reduction in internalized protein in presence of BT 3033 (# *p* < 0.001, n = 3). G) Control and integrin β1 shRNA-treated HK-2 cells were compared to estimate the amount of spike protein internalized. There was a significant reduction in internalized spike protein in the integrin β1 shRNA-treated cells compared to control shRNA-treated cells (# *p* < 0.001, n = 3).

The functional importance of inhibiting the activation of integrin α2β1 on the functions of ACE2-mediated cells was also studied. Based on recent studies that ACE2 and integrin β1 are important for SARS-CoV-2 internalization, the ability of spike protein endocytosis was investigated in the presence of integrin β1 inhibitor. In BTT 3033 treated cells, internalization of spike protein was significantly lower compared to DMSO treated cells (Fig. 5A, B). Similar results were obtained by the treatment of Caki-1 cells with BTT 3033. Control protein internalization was not altered with BTT 3033 treatment in both cell lines indicating the specificity of integrin α2β1-ACE2 complex in SARS-CoV-2 spike protein internalization. This was further confirmed with ELISA experiments that show significant decrease in the internalization of spike protein in the presence of inhibitor in HK-2 (Fig. 5E) and Caki-1 (Fig. 5F) cells. In addition, the integrin β1 KD cells treated with spike proteins also showed significant reduction in the internalization of spike protein compared to control shRNA treated cells (Fig. 5G).

### 3.6. Integrin β1 regulates ACE2 expression in the kidney epithelial cells

**Fig. 6.**
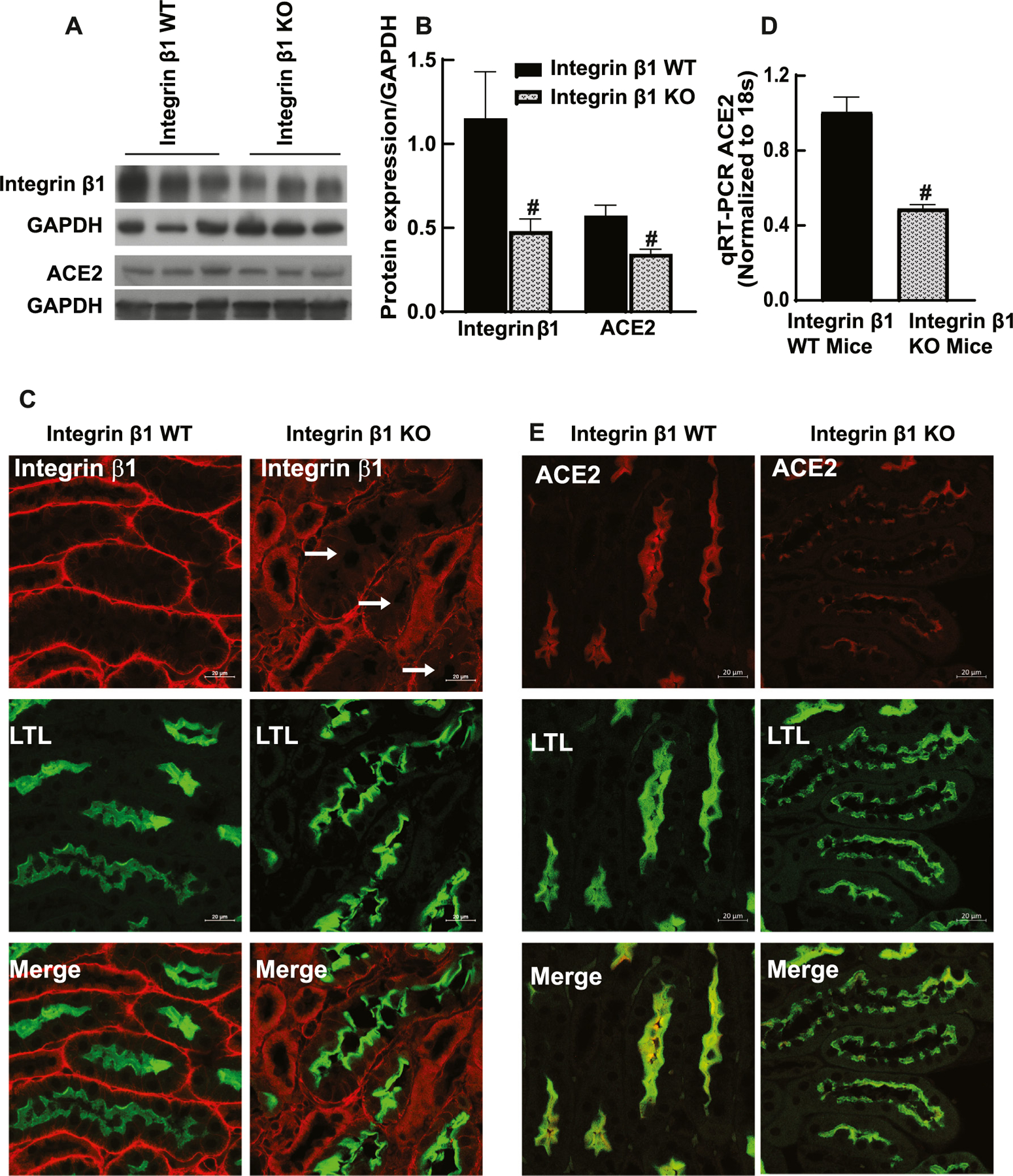
Deleting integrin β1 from the kidney proximal tubules epithelial cells decreased ACE2 expression. A, B) Renal proximal tubule-specific depletion of integrin β1 was carried out using a ɣgt cre. The decreased expression of integrin β1 was confirmed by immunoblotting of cortical lysate integrin β1 (Santa Cruz Biotechnology, SC-374430). Integrin β1 KO mice showed a significant decrease in expression compared to integrin β1 WT mice (# *p* < 0.05, n = 3). C) The epithelial cell-specific deletion was further confirmed by immunofluorescence. LTL (green) indicates the proximal tubules. Integrin β1(Abcam, ab179471) (red) staining was significantly reduced in LTL-positive cells. A, B) Immunoblotting shows a significantly lower ACE2 (Invitrogen, MA532307) in the kidney cortex of integrin β1 KO mice compared to WT mice (# *p* < 0.05, n = 3). D) mRNA analysis by qRT-PCR confirmed the decreased ACE2 expression. E) ACE2 expression in the proximal tubules was investigated using immune fluorescence. Proximal tubule marker LTL (green) was also used to counter-stain. Representative results from 3 independent mice kidneys show lower ACE2 (red) in the proximal tubules of the integrin β1 KO mice compared to WT mice.

The importance of integrin β1 on ACE2 levels of renal epithelial cells was investigated using in vivo models. Integrin β1 was deleted form the proximal tubules of the kidney using ɣ-gt cre. The decrease in integrin β1 levels were confirmed by immunoblotting of kidney cortical lysate (Fig. 6A, B). The efficiency and specificity of the cre was determined by using immunoblotting and immunofluorescence (Fig. 6C). With the reduced integrin β1 levels, the ACE2 expressions were decreased significantly in the kidney cortical lysate by immunoblotting (Fig. 6A, B *p* < 0.05). The mRNA levels were also decreased significantly by the absence of integrin β1 (*p* < 0.05, Fig. 6D). The epithelial cell specific decrease in the ACE2 was confirmed by immunofluorescence (Fig. 6E). Proximal tubule segments were counter-stained with LTL. These data confirmed that decreased expression of integrin β1 can reduce ACE2 expression in the proximal tubule cells of mice, which confirms our observation using kidney epithelial cells and cancer cells.

## 4. Discussion

Integrins are the major transducers of cellular micro-environmental changes to intracellular signaling pathways. The reciprocal communication between cells and their microenvironment is mediated by integrin’s association with multiple cytosolic and transmembrane proteins. The various intermediate proteins that regulate integrin mediated cell signaling are not completely identified. Results from this study suggest that integrin β1 activation modulates the ACE2 expressions in kidney proximal tubule epithelial cells. This study also suggests that the lower expression of integrin β1 leads to reduced ACE2 expression in the kidney, based on molecular knockdown, pharmacological and gene-knockout approaches. Importantly, this study for the first-time reports that integrin β1 is required to maintain adequate ACE2 expression for the entry of SARS-CoV-2 spike protein in renal tubular epithelial cells.

ACE2 regulates blood pressure, electrolyte balance, inflammation, oxidative stress and renal fibrosis through generation of angiotensin 1–7 and by decreasing angiotensin II levels,(Liu et al., 2017; Liu et al., 2012; Mizuiri and Ohashi, 2015). This study provides several lines of evidence for a regulatory role of integrin β1 in ACE2 expression in human and mouse renal epithelial cells. A ShRNA-mediated decrease in the expression of integrin β1 lowered ACE2 gene and protein expressions. Previously we demonstrated that a decrease in integrin β1 protein levels lowers the expression of multiple integrin α subunits(Mia et al., 2021). The major collagen receptor present in kidney cells is integrin α2β1. A selective pharmacological inhibitor of integrin α2β1 (BTT 3033), showed similar results(Li et al., 2021; Mogami et al., 2018). Integrin β1 expression is upregulated in multiple solid tumors and promotes tumor growth through various mechanisms including the regulation of fibrosis. Our data confirmed that the expression of integrin β1 is higher in renal tumor samples compared to normal tissue. This was further recapitulated in cancer cell models. These data indicate that an increase in integrin β1 might increase ACE2 expression in various kidney diseases. Furthermore, the use of integrin α2β1 inhibitor BTT 3033 significantly reduced ACE2 expression in renal cancer cells similar to that in epithelial cells. These findings suggest a correlation between integrin β1 levels and ACE2 in human kidney epithelial cells.

Even though the principal function of integrins is to mediate cell adhesion to the matrix protein in the basement membrane, studies have demonstrated an interaction with growth factor receptors in determining the specificity of signaling pathways. Integrins are known to associate with multiple receptor tyrosine kinases including EGFR, which is important in, tissue development, tumorigenesis, and fibrosis. Recently it is reported that EGFR activation could promote integrin tension and lower the threshold for focal adhesion and cytoskeleton organization resulting in a higher cell spreading (Rao et al., 2020). Reciprocally, antibody-mediated inhibition of integrin β1 decreased the total and activated EGFR protein in EGFR overexpressing breast cancer cells (Wang et al., 1998). The regulation by ACE2 also regulates the EGF transactivation in diabetes and phosphorylation of EGFR in kidney cells (Akhtar et al., 2012; Yosypiv et al., 2006). Recently, it is demonstrated that the binding of spike proteins to the cells activates multiple downstream signaling pathways including AKT and ERK1/2 through EGFR activation (Palakkott et al., 2022). In addition, the inhibition of downstream signaling pathways of EGFR efficiently inhibits SARS-Cov-2 viral replication in cells (Klann et al., 2020). Thus, the inhibition of spike protein internalization observed in this study might be a combined effect of the downregulation of ACE2 and other signaling cascades in the cells due to the integrin inhibition.

This study also suggests the importance of collagen I receptor integrin α2β1 in regulating ACE2 expression. ACE2 mediates the production of Ang 1–7 peptide from Ang II. The anti-fibrotic, cardio-protective and reno-protective effects of Ang 1–7 are well documented (Cassis et al., 2019; Morganstein et al., 2021; Simoes and Teixeira, 2016; Tao et al., 2014). Since integrin α2β1 is anti-fibrotic in nature (Agarwal et al., 2020; Kozlowska et al., 1996; Xia et al., 2012), the activation of integrin α2β1 might decrease fibrosis in multiple kidney diseases, most likely via maintaining ACE2 expression. This study has not explored the influence of integrin α2β1 activation on ACE2 mediated anti-fibrotic effects. Our study suggests that it is worth exploring the effect of integrin α2β1 in fibrosis and hypertension using various models of chronic kidney disease in the future.

Multiple reports have highlighted the role of ACE2 in SARS-CoV-2 viral entry (Shang et al., 2020b; Sigrist et al., 2020). The role of integrin β1 and its association with ACE2 in promoting SARS-CoV-2 has also been reported (Kliche et al., 2021; Makowski et al., 2021; Yan et al., 2020b). Therefore, our studies investigated the role of integrin β1 on the endocytosis of SARS-CoV-2 to kidney epithelial and cancer cells. Pharmacological inhibition of integrin β1 decreased the entry of SARS-CoV-2. We show that by altering the activation status of integrin α2β1 with a small molecule BTT 3033, the SARS-CoV-2 spike protein endocytosis can also decrease. This was also confirmed using two different cell lines, renal epithelial cells and renal cancer cells. These data suggest the importance of exploring the possibility of repurposing the FDA approved small molecule integrin inhibitors and antibodies to prevent SRAS-CoV-2 associated renal functional decline and renal injury in patients with kidney diseases.

Finally, the importance of integrin β1 in ACE2 expression was investigated using an in vivo model. The deletion of integrin β1 from the proximal tubule epithelial cells of kidney significantly reduced ACE2 expression. The mouse model used here achieved near complete deletion of the integrin β1 from the proximal tubule epithelial cells. Kidney cortical lysate contains multiple cell types, thus a total loss in integrin β1 expression was not observed. However, a significant decrease in the protein levels were found by immunoblotting of kidney lysate. The epithelial specific integrin β1 deletion was confirmed by immunofluorescence. A concomitant decrease in the ACE2 expression in the renal epithelial cells was confirmed by immunoblotting, qRT-PCR and immunofluorescence. Many transcriptional and epigenetic factors were identified in the regulation of ACE2 expression, which appear to be largely cell type-dependent. Hepatic nuclear factor 1α (HNF1α) and (HNF1β) are shown to increase the ACE2 levels in human embryonic kidney cells and pancreatic islet cells (Pedersen et al., 2013; Senkel et al., 2005). Integrins are not shown to regulate the HNF levels in these cells and thus may not be a potential contributor to integrin β1 mediated ACE2 regulation. Short-term hypoxia increased the transcription and translation of ACE2 in human hematopoietic stem cells and pulmonary artery smooth muscles however long-term hypoxia decreased the expression in the latter (Joshi et al., 2019; Zhang et al., 2009). These studies suggested that regulation of ACE2 expression by hypoxia-inducible factor 1α (HIF1α) is time-dependent. HIF-1α is also important in the regulation of integrin expression by directly binding to the promoter region (Goggins et al., 2021; Keely et al., 2009; Mendonca and Soliman, 2020). In addition, the Nuclear Factor Erythroid-Derived 2-Related Factor 2(Nrf2) downregulated the ACE2 expression in the alveolar epithelial cells whereas the inhibition of Nrf2 upregulated ACE2 in immortalized RPTCs (Mendonca and Soliman, 2020; Zhao et al., 2018). PI3kinase/AKT and MAP kinases are upstream regulators that activate the Nrf2 (Pan et al., 2017; Tsai et al., 2013). Since previous studies demonstrated that integrin β1 deletion downregulates the above-mentioned signaling pathways therefore very unlikely to be involved in the regulation of ACE2 by integrin β1. Furthermore, the circulating miRNAs miR-143 and miR-421 decreased ACE2 in leukocytes and cardiac myofibroblasts, respectively (Lambert et al., 2014; Trojanowicz et al., 2019). A positive feedback mechanism mediated by Ang II induced ACE2 shedding by promoting TNF-α converting enzyme activity (Patel et al., 2014). Transforming growth factor β1 (TGF-β1) is another factor reported to decrease the expression of ACE2 in renal epithelial cells (Chou et al., 2013). Collectively, these studies imply that transcriptional and post-translational mechanisms of ACE2 expression are complex and that focused study is required to elucidate molecular mechanisms of ACE2 regulation by integrin β1. In conclusion, this study discovered a new role of integrin β1 in kidney epithelial cells. By demonstrating the relationship between integrin β1 and ACE2 in the kidney, we show a major protein-protein regulation that has critical pathological implications in kidney diseases.

### 4.1. Limitation of the study

Since multiple integrin β1 mediated signaling pathways are dysregulated in many chronic diseases including hypertension and chronic kidney diseases, its effect on ACE2 levels is essential to establish the role of this protein-protein regulation on pathophysiology. In addition, integrin is known to associate with multiple receptor tyrosine kinases including EGFR. ACE2 also regulates the EGF transactivation in diabetes and phosphorylation of EGFR in kidney cells (Akhtar et al., 2012; Yosypiv et al., 2006). Thus, the inhibition of the ACE2 observed by the integrin β1 downregulation in this study could be a secondary effect of growth factor mediated cells signaling that needs further investigation and this study has not addressed the effect of different concentration of EGF on ACE2 expression. Moreover, studies are also needed to determine the existence of any reciprocal regulation of integrin β1 by Ang 1–7 mediated signaling. It is possible that there are several mechanisms that control ACE2 expression in cells and these causal-effect relationships are out of the scope for this manuscript and will be investigated in the future.

## Data Availability

Data will be made available on request.
